# Virtue Existential Career Model: A Dialectic and Integrative Approach Echoing Eastern Philosophy

**DOI:** 10.3389/fpsyg.2016.01761

**Published:** 2016-11-14

**Authors:** Shu-Hui Liu, Jui-Ping Hung, Hsin-I Peng, Chia-Hui Chang, Yi-Jen Lu

**Affiliations:** ^1^Department of Guidance and Counseling, National Changhua University of EducationChanghua, Taiwan; ^2^Chinese Career Research Center, National Changhua University of EducationChanghua, Taiwan; ^3^Department of Counseling Psychology, Chinese Culture UniversityTaipei, Taiwan; ^4^Department of Psychology, Fu Jen Catholic UniversityXinbei, Taiwan; ^5^Bai-Du System Design Co. LtdTaipei, Taiwan; ^6^Tainan Municipal Fusing Junior High SchoolTainan, Taiwan

**Keywords:** career theory, postmodernism, dialectical philosophy, virtue psychology, existential therapy, indigenous psychology, Chinese philosophy, *Classic of Changes (Yi-Jing)*

## Abstract

Our Virtue Existential Career (VEC) model aims at complementing western modernism and postmodernism career theories with eastern philosophy. With dialectical philosophy and virtue-practice derived from the *Classic of Changes*, the VEC theoretical foundation incorporates merits from Holland typology, Minnesota Theory of Work Adjustment, Social Cognitive Career Theory, Meaning Therapy, Narrative Approach Career Counseling, and Happenstance Learning Theory. While modernism considers a matched job as an ideal career vision and prefers rational strategies (*controlling and realizing*) to achieve job security; postmodernism prefers *appreciating and adapting* strategies toward openness and appreciates multiple possible selves and occupations, our model pursues a blending of security and openness via *controlling-and-realizing* and *appreciating-and-adapting* interwoven with each other in a dialectical and harmonious way. Our VEC counseling prototype aims at a secular goal of *living on the earth with ways and harmony* (安身以法以和) and an ultimate end to spiral up to the *wisdom of living up to the way of heaven* (天道) *with mind and virtue* (立命以心以德). A VEC counseling process of five major career strategies, metaphorical stories of *qian* and *kun*, and experiential activities are developed to deliver VEC concepts. The VEC model and prototype presented in this research is the product of an action research following Lewin's (1946) top-to-down model. Situated structure analyses were conducted to further investigate the adequacy of this version of VEC model and prototype. Data from two groups (one for stranded college graduates and the other for growing college students) revealed empirical supports. Y*ang* type of career praxes tends to induce actualization, which resulting in realistic goals and concrete action plans; *yin* type of career praxes tends to increase self-efficacy, which resulting in positive attitude toward current situatedness and future development. Acceptance and dialectic thinking often result from *yin*-y*ang-*blending career praxes. Growing developers benefit from a strategy sequence of *yang-yin*-synthesized; stranded developers from a strategy sequence of *yin-yang*-synthesized. Our contributions and limitations are discussed in the context of developing indigenous career theories and practices for a globalized and ever-changing world.

Careers should always be seen as embedded in societal, political, and economic contexts (Collin and Young, 1986) and shaped by chance events (Bright et al., 2009). Nowadays, a critical challenge for career practitioners and scholars is to answer “how people live a summative life in such a globalized and ever-changing world.”

The key to this question lies in the conceptual framework of career views. A career view reflects how an individual views and copes with his or her career issues, and it consists of career visions and projecting strategies (Liu, 2005). A career vision identifies WHAT one wants in one's career. One needs to figure out a meaningful and feasible life for one to live up to in one's world. A projecting strategy, as a way of *being-in-the-world*, identifies HOW one gets what one wants or takes what one is given or encounters. It also indicates the ways to live up to one's career vision. Everyone has his or her own career view, which indicates his or her ideal career evolution and favored career evolution strategies. Furthermore, each career theory reveals a kind of career view underlying its theoretical arguments.

In western literature, modernism and postmodernism career theories have proposed two types of answers (career views) to the question of summative life: settling down or opening up (Chudzikowski, 2012). Table 1 summarizes the comparison of these two types of answers.

**Table 1:** **The comparison of modernism and postmodernism career approaches**.

**Career**	**Modernism**	**Postmodernism**
**CAREER VISION**		
Targets	One matched occupation	Multiple possible selves and occupational options
Reactions to uncertainty	Controlling for security	Adapting to change
Ideal status	Actualizing stable personal characteristics within congruent environment	Constructing individual identity within varying contexture
Expected Outcomes	Occupational success and goal fulfillment	Life satisfaction and meaning
**PROJECTING STRATEGY**		
Archetype of strategy	Choosing and controlling	Appreciating and adapting
Functioning mechanism	Rational and analytic thinking	Intuitive and expressive thinking
Self-exploration method	Objective quantitative assessments	Subjective qualitative assessments
Environmental exploration method	Non-involved information processing	Involved information experiencing
**CAREER EVOLUTION**		
Nature of person and environment	Static and stable	Dynamic and varying
Evolution styles	Focus and controlling	Open and appreciating
Evolution pattern	Linear	Non-linear

Modernism (Chartrand, 1991; Kristof-Brown et al., 2005; Sampson, 2009) pursues the goal of settling down. It prefers a matched job and a linear sequence of employment-related positions, roles, activities, and experiences, which share common characteristics and cumulate occupational success and satisfaction. Change and career uncertainty are considered as something bad and people are encouraged to catch the constant parts out of changing lives. With the stress of instrumental rationality and subjectivity, the modernists rely on rational strategies (*controlling and realizing*, 掌控落實), which emphasize analytic thinking to capture a precise understanding of self and environment, to achieve job security. It values standardized quantitative assessments and aggregate factual career information.

Such a paradigm definitively has its merits; which is why it could survive for more than one century. Nevertheless, more and more western postmodern career theories have emerged to echo Trevor-Roberts's (2006) calls for positive reactions to career uncertainty. Gelatt's positive uncertainty (Gelatt, 1989, 1995), Krumboltz's happenstance learning theory (Mitchell et al., 1999; Krumboltz, 2009), and Pryor and Bright's career chaos theory (Pryor and Bright, 2003, 2006, 2007) are three of the most popular ones. These theories consider change and uncertainty as something good and people are encouraged to embrace the inconstant nature of lives. They advocate a life of opening up to multiple possibilities. With the concern of synchronicity and intersubjectivity, intuitive and expressive strategies (*appreciating* and *adapting*, 悅納變通) are provided for an experiential understanding of self and environment. As a result, the postmodernism summative career evolution may be a combination of employment-related or unrelated positions, roles, activities, and experiences, which may possess objectively different characteristics and subjectively constructed fulfillment and meaning.

The purpose of this research is to propose the third type of answers (career views) about a summative life, which complements western modernism and postmodernism career theories with ancient Chinese philosophy from the *Classic of Changes* (易經, an ancient divination text, also known as the *I Ching*, the *Book of Changes*, the *Zhouyi* or *Zhou Changes*). Such an ancient Chinese wisdom has been considered as the very essential of Chinese culture as well as a world philosophy which is rooted in the universal humanity and appeals to entire human being (Hwang, 2009; Hwang and Chang, 2009; Cheng, 2013; Liu, 2014). The Virtue Existential Career Model (Liu and Wang, 2014; Liu et al., 2014, 2016; VEC model, Liu et al., 2015) uses the *Classic of Changes* and its subsequent Confucian and Taoist philosophies to address ordinary people's practical and existential career concerns. Such a model echoes Hwang's (2012) advocacy for culture-inclusive psychology as well as Liu et al. (2008) proposition for social and cross-cultural psychology as a global enterprise.

Moreover, echoing pluralism advocated by postmodernism, the VEC model intends to incorporate merits from both modernism and postmodernism career views. Martin and Sugarman (2000) have articulated a middle-ground position between modernity and postmodernity; Casey (2000) has called for a revitalized project to transcend the limitations resulted from modern or postmodern approaches alone; Zhao (2007) has argued that it takes the postmodern value of intersubjectivity and mutual connection to nurture one's inner self and, therefore, make a modern subject without alienation. Still more, Sampson (2009) has pointed out directly that career practitioners can provide better services if modern and postmodern career theories go hand in hand with each other. Hence, our model pursues an enjoyable blending of security and openness via career evolution resulting from *controlling-and-realizing* and *appreciating-and-adapting* interwoven with each other in a dialectical and harmonious way.

This paper presents the VEC model in three perspectives: (1) its theoretical foundation, (2) its counseling prototype, and (3) situated structure analyses of two VEC group experiences.

## Theoretical foundation

While modernism career paradigm is based on positivism and postmodernism career paradigm is derived from constructivism and contextualism (Sampson, 2009), the VEC model has its root in Chinese philosophy with its branches from modernism and postmodernism career theories and practices.

We will first introduce dialectical philosophy and virtue practice derived from the *Classic of Changes*. Both their original contentions and their applications in west psychology will be presented, followed by their applications to our model. Then, we will describe what and how our model adopts modernism and postmodernism theories. In addition, we also present what and how we adopt existential-phenomenological psychology and meaning therapy.

### Eastern wisdom: dialectical philosophy

The *Classic of Changes* articulated a cosmological model of changes via the fluxion of *yin* (陰) and *yang* (陽). Every element in every system of this universe is composed of these two essentials. *Yin* (literally, dark) represents *receptivity* and denotes the power of earth; *yang* (literally, light) represents *creativity* and denotes the power of heaven. Actually, *yin* and *yang* symbolize all kinds of dyadic opposites: anything relatively hard, high, light, strong, firm, moving, flow, noble is considered as *yang*, and anything relatively soft, low, heavy, weak, flexible, static, stuck, mean as *yin*. Dyadic opposites are not separable but rather form a unity together. Such *yin-yang* fluxion creates and transforms all things existing in between earth and heaven (Vincent Shen, 2003; Cheng, 2009a,b).

The principles underlying the *yin-yang* fluxion are basically dialectical, such as *mutual completion and enhancement* (相生相成), *generation by opposition* (對立轉化), and *joint production* (生生不息) (Cheng, 2009a; Liu et al., 2015). Harmony is the shared aim of these principles, which is signified in the opening passages of the *Classic of Changes* (Nelson, 2011). Through harmonizing, elements contradict and yet mutually supplement each other and thereby build a new relation or form a new unit (Vincent Shen, 2003; Cheng, 2009b; Yao, 2013). Such *yin-yang* fluxion entails a sustained and dialectical process as a temporally successive phenomenon with no linear theory of causality.

A similar dialectic philosophy has been articulated by Hegel, a universally influential philosopher (Brincat and Ling, 2014). This kind of dialectic thinking has been adopted by western psychological theories, such as Jung's Analytical psychotherapy (Solomon, 1994; Liang, 2012), dialectical behavior therapy (Bankoff et al., 2012; Linehan and Wilks, 2015), second wave positive psychology (Wong, 2011, 2012; Lomas and Ivtzan, 2015), and contextual action theory for career development (Dyer et al., 2010). Actually, more and more western research and theories have highlighted a dialectical nature of various psychological phenomena, such as human development (Komatsu, 2015) and creative process (Holm-Hadulla, 2013).

Based on all the above, our model applies this dialectic philosophy to career evolution and counseling. *Appreciating*-and-*adapting* types of career projecting are considered as y*in*; *controlling-and-realizing* types of career projecting as y*ang* (Liu et al., 2015). Career development is a harmonizing process with a continuous and discontinuous interplay of preceding and consequent moments in time. Such a career evolution process is different from the traditional linear pattern advocated by the abovementioned modernism career paradigm; rather, it is closer to a *boundaryless or protean* pattern (Stoltz et al., 2013) described by the abovementioned postmodern career theories.

Just as *inclusive opposites* is the power and energy of the harmonizing process, career explorers who flexibly utilized both types has been found to demonstrate more resilience, passion, and well-being (Lee, 2010; Kao and Liu, 2012; Peng et al., 2013). Empirical supports also revealed that the Middle Way (中庸, accepting the coexistence of *yin* and *yang* and emphasizing harmony) beliefs positively associated with life satisfaction (Huang et al., 2012) and mitigated the effects of stress on employee well-being and job satisfaction (Chou et al., 2014).

### Eastern wisdom: virtue-practice as self-cultivation

There are many branches of interpretation; Confucian and Taoist philosophies are two of the most influential ones (Hwang and Chang, 2009). Confucian interpretations of the *Classic of Changes* are primarily ethical (Yao, 2013). Confucians take one's *de* (德, virtue) and cosmological *tao* (道) as the ultimate concerns of life meaning. The self is considered both the object that must be worked on, and the subjective agent that bears chief responsibilities for the well-being of oneself, one's family, and the world. Working on the self is to cultivate it, which undergoes a moral process starting from an ordinary person and reaching the ideal (Hwang, 2009; Yao, 2013).

Parallel to ethical living and self-cultivating advocated by Confucian in the east is “being-in-the-world-with-others” or “relatedness” advocated by phenomenology in the west (Becker, 1992). Phenomenologists consider humans as inherently relational beings. “We are summoned ethically to cultivate our way of interrelating so as to serve others and the non-human natural community (Adams, 2007, p. 24).” Such a point of view has been brought into psychology and psychotherapy (Becker, 1992). Similarly, contextual action theory considers a career as a joint parent–youth project. That is to say, career is embodied in how youth relate to others (Young et al., 2008).

Taoist interpretations of the *Classic of Changes* are primarily authentic (Hwang, 2009). Taoists seek to emancipate their selves from the ethical bounds of a societal world and follow the way of Nature (Karcher, 1999). By practicing methods of self-cultivation, such as *qi-gong, tai-qi-chuan*, and Taoist meditation, one may return to the state of authentic self (Hwang, 2009).

Taoist interpretations of the *Classic of Changes* were first introduced to western psychology by Jung. His three principles of psyche actually echo Taoism (Karcher, 1999; Liang, 2012). Taoist techniques have also been integrated into Jung and other psychological therapy models with empirical supports (Williamson, 1992; Hwang, 2009).

Recently, two kinds of voices pro virtue emerge in western psychology. One voice came from positive psychology. Sundararajan (2005) pointed out that “an empirically based version of the good life as proposed by positive psychology is a donut with something missing at the core—the moral map (p. 35).” To make an amendment, Wong proposed Positive psychology 2.0 and advocated meaning-orientation visions of a good life (2011) and self-transcendence (2016). The other voice argued psychotherapy is a moral encounter (Burns et al., 2012). A tradition-sensitive psychotherapy should function emically within the client's own virtue grammar (Dueck and Reimer, 2003; Peteet, 2013) and help clients cultivate the qualities necessary to live a good life (Stewart-Sickin, 2008).

Based on all the above, our model emphasizes virtue-practice in career evolution and counseling. An ideal aim of one's career evolution should be two-folded:.(1) a *yin* horizon of *living on the earth with ways and harmony* (安身以法以和) and (2) a *yang* horizon of *living up to the way of heaven with mind and virtue* (立命以心以德) (Liu et al., 2014, 2015, 2016; Liu and Wang, 2014). Therefore, an ideal career evolution is an endless process of self-cultivating, that is, living a moral life while being entangled by worldly affairs and struggling for *living on the earth*.

The two horizons can be articulated with the terminology of Hwang's (2012) mandala model of self. *Living-on-the-earth-with-ways-and-harmony* is related to one's career praxes to find a place in real world to fulfill one's biological needs (as individual), to find a position in social networking to carry out one's social responsibilities (as person), and to establish an identity to actualize one's psychological characteristics (as self). One's knowledge might be used to exchange various resources in one's career pursuing. A morally cultivated person seeks good from others and at the same time exerts good effects on these others, by which a harmonious relationship is established (Hwang, 2009; Yao, 2013; Liu, 2014).

*Living-up-to-the-way-of-heaven-with-mind-and-virtue* points to one's wisdom and self-cultivation to become a Self. It assumes that one's self needs to be created, developed, and realized in the ethical life while the potentiality of building a self is given (Chen, 2014). Specifically, one is expected to possess a balance between *yin* virtue (embracing all aspects of humanity and accommodating all matters in this world, 厚德載物) and *yang* virtue (unceasingly striving for improving themselves with their great perseverance, 自強不息; Cheng, 2009a; Liu et al., 2015). Such wisdom helps one adapt to the inevitable uncertainty within career evolution and appreciate the underlying *way-of-heaven* within each life experience.

### Western wisdom: career vision

There are three key questions about career vision. (1) What does one want from the world? (2) What can one supply to the world? (3) Why should one live one's life?

As to one's wants in career vision, we adopt Holland's interest (Nauta, 2013) and Minnesota's needs and work values (Swanson and Schneider, 2013). Vocational interests are defined as “a preference for activities expressed as likes and dislikes” (Hansen, 2013, p. 387). Holland's theory focuses most explicitly on interests and its RIASEC typology pervades career counseling research and practice (Nauta, 2010). Several meta-analyses have supported the existence of the RIASEC typology among a variety of people (Nauta, 2010), the stability of vocational interests (Low et al., 2005), and the predictability of congruence on career choice (Sheu et al., 2010), stability of choice (Spokane, 1985), academic performance and persistence (Spokane, 1985), job satisfaction, job performance, and turnover (Tsabari, 2005; Van Iddekinge et al., 2011).

According to Minnesota Theory of Work Adjustment (TWA), vocational needs refer to what one wants and expects from work; work values are defined as “second-order needs” or “underlying common elements of needs.” TWA's 6-value and 21-need classification is adopted by O^*^NET and becomes one of the most important value system (Rounds and Jin, 2013; Swanson and Schneider, 2013). Meta-analyses revealed value congruence was positively related to work satisfaction, overall work performance, and organizational commitment, as well as negatively related to turn-over intention (Kristof-Brown et al., 2005).

As to one's supplies in career vision, we adopt self-efficacy from Social Cognitive Career Theory (SCCT) (Lent, 2013). According to Bandura (1986, p. 391), self-efficacy beliefs refer to “people's judgments of their capabilities to organize and execute courses of action required to attain designated types of performances.” Although, TWA emphasizes on one's ability to respond to environmental requirements (Swanson and Schneider, 2013), SCCT argues that self-efficacy, as subjective assessment of ability, is a better predictor of career development. Meta-analyses indicated that self-efficacy beliefs correlated significantly with academic choice, performance, and persistence (Brown et al., 2008; Sheu et al., 2010), vocational choice and work-related performance (Sheu et al., 2010; Brown et al., 2011).

As to one's meaning in career vision, Frankl (1992) asserts that the deepest and universal human need to reach beyond oneself and serve something greater. Along with this line of think, Kosine et al. (2008) proposed purposes; MacIntyre (1984, cited in Stewart-Sickin, 2008) proposed virtues. Wong (2016) takes one step further and suggests three levels of self-transcendence, which can be summarized as ultimate meaning, situational meaning, and one's calling. On the other hand, Cochran (1997) suggests that one explores the implicit personal webs of meaning embodied within narrative scripts or plots. The fulfillment of meaning is related to greater certainty regarding career goals (Tryon and Radzin, 1972) and better work adjustment (Bonebright et al., 2000).

### Western wisdom: projecting strategy

Key questions to projecting strategy are the following. (1) How does one construct and reconstruct one's career vision in one's career evolution? (2) How does one live up to one's career vision?

We adopt Holland's (as well as other modernism theories of TWA and SCCT) *yang* type of strategies to: (1) use quantitative assessments to identify one's interest (or ability, need, or self-efficacy) pattern, (2) develop a list of educational and/or occupational options congruent with one's interest pattern, (3) search for and process factual educational and/or occupational information, (4) choose an educational and/or occupational goal based on rational evaluation, (5) search information about resources and strategies helpful for one's specific goal, and (6) break one's final goal into sub-goals and set up a step-by-step action plan to carry out those sub-goals (Nauta, 2013).

We also adopt Cochran's (1997) *yin*-*yang*-mixed type of postmodernism strategies to: (1) enhance a sense of agency in reality construction; (2) gain information from divergent sources and to gain the best evidence; (3) envision oneself working in an occupation; (4) identify needs and establish a tentative priority; (5) determine strengths in the way an individual and family function to get things done; (6) map the formal and informal sources of support that might help a person solve a problem or move from a deficient state of affairs to a better state of affairs; (7) establish an optional alignment of needs, strengths, supports, and resources to meet needs; (8) actualize ideals in the present through searching for and engaging in activities that are meaningful and enjoyable.

Finally, we learn from Krumboltz's postmodernism strategies of treasuring serendipity and uncertainty. At first, Mitchell et al. (1999) brought out five *yin* type of planned happenstance skills: Curiosity refers to exploring new learning, persistence to exerting efforts in spite of setbacks, flexibility to adjusting to change, optimism to believing in new opportunities, and risk-taking to taking action in spite of uncertainty. Positive relationship between these skills and career evolution has been found (e.g., Magnuson et al., 2003; Kim et al., 2014).

Later, the revised Happenstance Learning Theory (Krumboltz, 2009, 2011; Krumboltz et al., 2013) proposed a counseling process with the following *yin*-*yang*-mixed type of strategies: (1) orienting the client to viewing unplanned events as necessary and normal to initiate a ready mode; (2) setting the goal of creating more satisfying lives to induce career action, (3) expressing empathic understanding of the client's situation and concerns to unfold a counseling process, (4) identifying the client's past success to strengthen self-efficacy and support current action, (5) assisting the client to capitalize on unplanned events for learning and exploration, (6) brainstorming the next action step with the client to produce desirable chance events; (7) helping the client to overcome blocks to action, (8) following up the client's action and reinforcing their success in the real world, and (9) in case of need, helping the client to overcome fear of making mistakes and to take action.

## The VEC counseling prototype

### A whole picture

**Figure 1:**
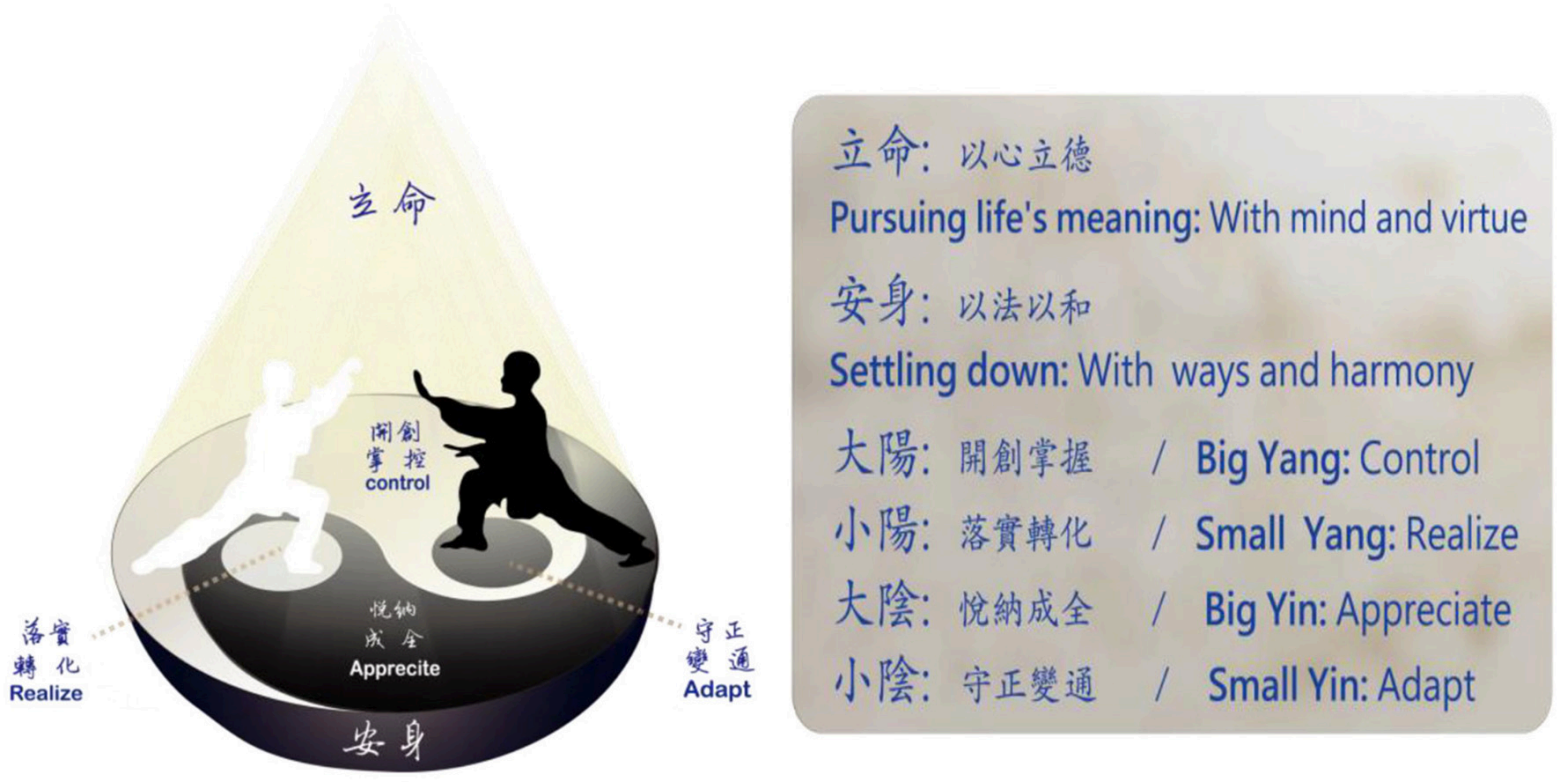
**Virtue existential career model**.

Figure 1 portrays our model with a cone. The *yin-yang tai-ji* (太極) diagram at the bottom of the cone displays the characteristics of *yin*-*yang* fluxion. *Yang* career strategy of *controlling and realizing* is symbolized by the white. The white body of fish on the upper part (*Controlling*) represents creative career praxes, that is, all kinds of directing and controlling toward one's career vision; the white eye of the black fish in the middle part (*Realizing*) represents a realistic effort to manage personal weakness or environmental requirements or restrictions. On the other hand, *yin* strategy of *appreciating and adapting* is symbolized by the black. The black body of fish on the lower part (*appreciating*) represents receptive career praxes, which result from appreciating whatever one is given or encounters; the black eye of the white fish in the middle part (*adapting*) represents a flexible effort to transfer one's capacities or environmental resources to find alternative ways for one's career vision in the reality. Career praxes, generated by the above four career strategies, serving as means for the secular goal of *living on the earth with ways and harmony*, construct the bottom of career evolution pyramids. However, the greatest end for career praxis is to spiral up to the *wisdom of living out of personal virtue*, which is congruent with cosmological *tao*.

Based on the *Classic of Changes*, it should function best when *Controlling, realizing, appreciating*, and *adapting* interweave with each other at a good timing and the *tai-ji* diagram becomes an unceasingly running circle in career evolution. *Realizing* and *adapting* in small circles in the middle part are career strategies with *yin*-*yang*-mixed transformative power. They play a critical role in connecting the pure *yin* power (*appreciating*) with pure *yang* power (*controlling*), and, in a paralleled or symbolic way, also connecting rationalism with constructivism, objectivity with subjectivity, the ideal with the reality, the heaven with the earth, as well as the wisdom (or virtue or self-cultivation) with the praxes.

### The outline of counseling prototype

Dialectical philosophy is applied to our prototype in four ways: (1) mixing *yin* and *yang* types of career strategies; (2) guiding people to appreciate, master, and synthesize both *yin* and *yang* types of career capacities and strategies; (3) guiding people to make connection between their secular and spiritual horizons; (4) using our metaphorical stories of *qian* and *kun* as well as creative activities to help people develop versatile perspectives.

Virtue-practice is embodied in the following VEC concepts: (1) the unceasing and dialectical nature of *yin-yang* fluxion in career and life; (2) the harmonious and integrative way of blending *yin* and *yang* career capacities and strategies; (3) the coexistence and mutual influence between secular and spiritual horizons within career evolution; (4) career evolution as a learning process to make a living, have a satisfying yet moral life, and cultivate wisdom and virtues.

**Figure 2:**
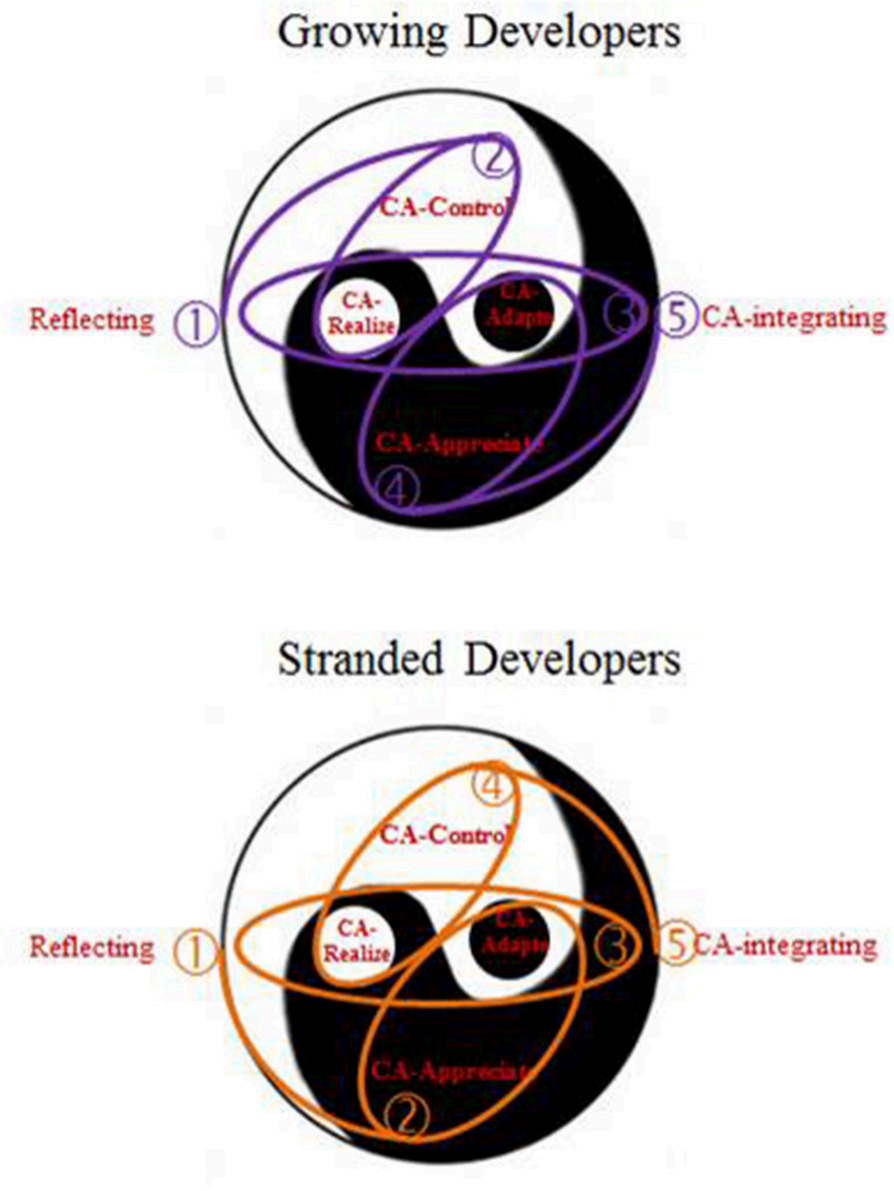
**Two sequences for growing and stranded developers**.

The VEC counseling process consists of five major career counseling strategies interwoven with each other in a dialectical and harmonious way. The five are CA-*controlling (yang), C*A-*realizing* (*yang* in *yin*), CA-*appreciating (yin)*, CA-*adapting* (*yin in yang*), and CA-synthesizing (*yang* with *yin*). Table 2 lists core and optional aims for each strategy. The arrangement principles are the followings. (1) A complete circle consists of a reflecting activity (to initiate a reflective attitude to find out underlying principles embodied within career phenomena) at the beginning, CA-synthesizing (to blend *yang* and *yin* power together) at the end, and the other four strategies in the middle. (2) For growing developer (those who are expecting to move on to the next developmental phase), CA-*controlling* or CA-r*ealizing* (to pursue what is wanted with or without consideration of environmental constraints) comes first; for stranded developer (those who are stuck in difficulties), CA-*appreciating* or CA-*adapting* (to accept what is given or encountered with or without consideration of one's wants) comes first. (3) CA-r*ealizing* and CA-*adapting* (both of which have a nature of blending of or transforming between *yin* and *yang*) are necessary between CA-*controlling* (pure *yang*) and CA-*appreciating* (pure *yin*). These three principles result in two possible sequences: for growing developer, a typical sequence may be: (1) reflecting, (2) CA-*controlling* or CA-r*ealizing*, (3) CA-*realizing* and CA-*adapting*, (4) CA-*appreciating* or CA-*adapting*, and (5) CA-synthesizing; for stranded developer, it may be: (1) reflecting, (2) CA-*appreciating* or CA-*adapting*, (3) CA-*adapting* and CA-*realizing*, (4) CA-*controlling* or CA-*realizing*, and (5) CA-synthesizing. Figure 2 visualizes these two routes.

**Table 2:** **The outline of VEC counseling prototype**.

**Career Strategies/Core Aims/Optional Aims/Possible Activities**
1. CS-Controlling (Yang) (1) Core Aims: Identify and create what one want via planning, directing, and controlling (2) Optional Aims: Identify what one want Count and make good use of whatever one has Create, construct, and present one's strengths Plan and take action to get more Overcome personal or environmental obstructs (3) Possible Activities Process assessments to identify what one want in terms of interests, values, aptitudes, and so on Process assessments to identify what one has in terms of knowledge, skills, and so on Process assessments to identify one's career capacities and strategies Outline, narrate or portray career vision Make an action plan to improve one's strengths Make an action plan to actualize one's career vision Actualize and follow up a career action plan
2. CS-Realizing (Yang in yin) (1) Core Aims: Make a realistic effort to manage personal weakness or environmental requires or restriction (2) Optional Aims: Understand and fulfill environmental requires Search for and utilize external information and/or resources Utilize one's power in a humble way Understand and manage one's situatedness (3) Possible Activities Reflect on qian stories Explore one's environments Practice information and/or resources searching Practice self-promoting skills Practice one's roles
3. CS-Appreciating (Yin) (1) Core Aims: Enjoy the nature of career evolution and whatever one is given or encounters (2) Optional Aims: Accept and make good use of whatever one is given or encounters Enjoy serendipity and uncertainty Learn from one's situatedness (3) Possible Activities Reflect on kun story Normalize, experience and treasure serendipity and uncertainty in life Develop versatile perspectives of oneself and one's surrounding Reflect on the nature of career with short films or imagination Reflect on personal experience and life meaning Portray or narrative one's experience and find out embodied values
4. CS-Adapting (Yin in yang) (1) Core Aims: Make a flexible effort to transfer one's capacities or environmental resources to find alternative ways for one's career vision in the reality (2) Optional Aims: Make creative use of whatever one is given or encounters Create alternative career possibilities in spite of constraints Be responsible for one's life meanings in spite of adverseness (3) Possible Activities Brainstorm alternatives Take advantages of serendipity Take advantages of frustration, difficulties, and obstructs Make a plan for alternative career possibilities in a creative way
5. CS-Synthesizing (Yang with yin) (1) Core Aims: Utilize both yin and yang power in a creative and harmonious way (2) Optional Aims: Synthesize opposites Synthesize both yin and yang types of career capacities and strategies (3) Possible Activities Practice brainstorming, creative art, or dialectical methods to synthesize opposites Practice synthesizing both yin and yang types of career capacities and strategies

### Activities and materials

Various career activities are developed to help developers learn VEC concepts in an experiential way. Our prototype incorporates *yang* type of career praxes (e.g., quantitative assessments, factual career information, rational evaluation, and action plans) and *yin* type of career praxes (e.g., normalizing and reframing unplanned events, identifying past success, and brainstorming the next action step). In addition, graphical materials, expressive therapeutic skills, guided imagination, mandala drawing, and meditation are adopted to stimulate right-brain functions and spiritual energy. These career praxes appear as possible activities in Table 2.

Specifically, our stories of *qian* and *kun* are designed to deliver the principles of *yin-yang* fluxion in a metaphorical way. The *Classic of Changes* consists of 64 *guas* (卦); a *gua* is a figure composed of six stacked horizontal lines (爻), where each line is either *yang* (an unbroken, or solid line) or *yin* (broken, or an open line with a gap in the center). Each *gua* symbolizes a common human life situation; its *yin-yang* fluxion denotes appropriate action strategies and self-cultivation goals within such situatedness. The *qian gua* with six solid lines represents a pure *yang* situation; the *kun gua* with six open line represents a pure *yin* situation. Variation of these two *guas* results in the other 62 *guas*. In other words, these two *guas* are the door to all varieties of life situations (乾坤易之門, Huang, 2003). We intended to make our metaphorical stories universally-applicable. Our *qian* story narrates six growing-up statuses of the protagonist dragon in sequence: (1) hidden to learn and improve oneself, (2) visible to be noticed and given opportunities or resources, (3) diligent to work hard in a mindful way, (4) jumping to try to leap up to a higher level, (5) flying to bring into full play, and (6) over-excited so as to exceed proper limits and result in regret. Our *kun* story narrates how the protagonist mare searches and serves a right master with six statuses in sequence: (1) cautious to watch out for any unremarkable sign for danger, (2) righteous to supports everything in a nature way, (3) dedicated to devote one's brilliance to the superiors without claiming credit for oneself, (4) invisible to hide one's candle and avoid making mistakes or threatening the superiors, (5) humble via doing influential work but adhering to one's subordinate role, and (6) arrogant so as to exceed proper limits and incur terrible danger. The *qian* and *kun* stories could be used in *yang* and *yin* types of strategies, respectively.

## Member experience of two VEC groups

### Research methods

The VEC model and counseling prototype presented in this research is the product of a previous action research following Lewin's (1946) top-to-down model with a cyclical-spiral process. In the planning stage, data from the third stage of the previous circle would be presented to a panel consisted of career experts and *Classic of Changes* experts. Based on the panel discussion, a revised VEC model and prototype would be proposed. In the action stage, groups would be conducted accordingly. During the evaluation stage, we conducted basic content analyses of observers' group process notes as well as products of and feedback from group members. Five versions of VEC model and prototype have been developed in this process from 2012 to 2015 (details reported in Liu and Hung, 2015). All studies conformed to the regulation of Research Ethics Committee of National Changhua University of Education.

The current study conduct situated structure analyses (Liu et al., 2016), a kind of phenomenological data analysis derived from Ricoeur (1981), Giogi (1989), and Gee (2005), to further investigate the adequacy of the fifth version of VEC model and prototype. Data from the third and fourth groups will be used. The third group was a 3-day 9-h group for 10 college graduates who had been seeking job without success and were referred by government's occupational placement service stations. The fourth group was a 3-day 18-h group for 8 recruited college students who were eager to prepare for future employment. Tables 3 and 4 present the designed activities and their corresponding projecting strategies, functioning mechanism, and member participations for these two groups.

**Table 3:** **VEC group design and member experience for growing college students**.

**Step**	**Projecting strategies**	**Functioning mechanism**	**Designed activities**	**Ms' Experience**
1	(−) *Appreciate*	(R+I) Conceptualize with films	**Constructing VEC concepts** (Day 1, 50′): L presented short films (*yin*) and delivered the following VEC concepts (*yang*).	Ms felt more comfortable with uncertainty.
2	(+) *Control*	(I+R) Portray + analyze	**Portraying a career vision and evaluating personal career readiness** (Day 1, 130′): Ms did a collage with mixed media (*yin*) to visualize their career visions. Ms rated their overall readiness for their expected visions (*yang*).	Ms demonstrated a better sense of what they wanted from the world and what they needed to supply to the world.
3	(+in−) *Realize*	(I) Narrative with our *qian* story	**Reflecting on our** ***qian*** **story** (Day 1, 50′): L presented pictures and narrated *qian* story (*yin*). L guided Ms to identify with the flying dragon.	Ms failed to identify with the flying dragon. They though adapting was more important than controlling.
4	(+in−) *Realize*	(R) Analyze	**Exploring the world of work** (Day 1, 130′): At first, Ms outlined (*yang*) their imagination about their ideal occupations. Then, Ms searched factual information (*yang*) via the internet.	Ms were impressed with the substantial differences found between imaginative and realistic versions of occupations.
5	(+) *Control*	(R) Analyze	**Identifying occupational capacities** (Day 2, 150′): Ms took the CCN skill Inventory (http://careering.ncue.edu.tw/style/?20) and compare their mastery levels with required levels of their targeted occupations (*yang*).	Ms were interested in test results and asked related questions.
6	(+) *Control*	(R+I) Plan with our mind map technique	**Making a learning plan to improve occupational capacities** (Day 2, 90′): With the technique of tree-like mind map (*yin*), for each skill, Ms listed possible usage in the future workplace or strategies to improve mastery in college (*yang*).	Ms went into details about their occupational capacities and learning plans.
7	(+in−) *Realize*	(I+R) Portray+ Analyze	**Practicing our “surviving game in a workplace”** (Day 2, 60′): Ms drew up their possible challenges in their future workplace (*yin*) and narrated about how they would use their skills to meet those challenges (*yin*).	Ms demonstrated confidence in their narratives.
*8*	(+in−) *Realize*	(R) Rehearsal	**Outlining resume with personalized occupational strengths** (Day 2, 30′): Ms outlined personalized occupational strengths for their resumes (*yang*). Relevant past experiences and potential applications in their future workplace were identified.	Ms revealed that previous activities helped them come up with concrete information to put on their resumes. They became more confident.
*9*	(+in−) *Realize*	(R) Rehearsal	**Practicing promoting personalized occupational strengths in job interviews** (Day 2, 40′): Each M took turns to go on stage and practice answering a typical job interview question of “why should I (boss) hire you” for 2 minutes (*yang*).	Ms reported learning in how to organize relevant information.
10	(−) *Appreciate*	(I) Narrative with our *kun* story	**Reflecting on our** ***kun*** **story** (Day 2, 30′): L presented pictures and narrated *kun* story (*yin*). Ms were encouraged to learn from *kun* mare.	Ms identified with the *kun* mare to calm down and use what they have creatively as well as improve what they need progressively.
11	(−) *Appreciate*	(I+R) Portray+ Analyze	**Constructing lived workplace strengths** (Day 3, 60′): After guided imagery of their past (*yin*), Ms drew a life line with critical events (*yin*) and narrated their associated workplace strengths (*yin*).	Ms found the VEC concepts of *mutual completion and enhancement* and *joint production* in their past life narratives and became more confident in their workplace adaptation and socialization.
12	(−in+) *Adapt*	(I+R) Portray+ Analyze	**Practicing “meaning-making game in life field.”** (Day 3, 60′): Each M took turns to add a gift, a challenge, and a comfort to other Ms' “surviving game in a workplace” pictures. Then, Ms utilized their lived strengths to make their disrupted pictures be their loved ones again.	The process of this activity was full of laughter and screaming. After reconstructing their pictures, Ms grasped the *generation by opposition* principle and the nature of harmonizing.
*13*	(+in−) *Realize*	(R) Rehearsal	**Writing resume with personal strengths** (Day 3, 60′): L gave a talk about the knack of resume writing (*yang*). Then, each M finished a complete resume (*yang*).	Ms got a better idea about what job interviewers wanted and improved their self-promoting skills.
*14*	(+in−) *Realize*	(R) Rehearsal	**Practicing a complete job interview** (Day 3, 80′): Ms practiced in pairs. Each M took turns to play the role of interviewee and boss (yang).	All “interviewees” did much better in their second practice.
*15*	(+with−) Synthesize	(R+I) Conceptualize with our VEC *tai-ji* diagram	**Constructing the VEC concepts of harmonizing with our VEC** ***tai-ji*** **diagram** (Day 3, 30′): L presented our VEC *tai-ji* diagram and articulated the VEC concepts of *yin-yang* fluxion. Then, L reviewed their group process and related it to these VEC concepts.	Ms mentioned that our VEC *tai-ji* diagram helped them develop a dialectic perspective of career evolution and a positive attitude toward whatever they might encounter.
*16*	(+with−) Synthesize	(I+R) Conceptualize with mandala drawing	**Practicing harmonizing with mandala drawing** (Day 3, 60′): Within a big circle (a mandala circle symbolizing Self), Ms synthesized all their positives and negatives into their ideal Self and career visions.	Most Ms presented their positive-negative-coexistence in a creative and harmonious way.

**Table 4:** **VEC group design and member experience for stranded college graduates**.

**Step**	**Projecting strategies**	**Functioning mechanism**	**Designed activities**	**Ms' Experience**
1	(−) *Appreciate*	(R) Conceptualize	**Constructing VEC concepts** (Day 1, 10′): L introduced VEC concepts in a way similar to the group of growing college students (*yang*).	Ms' faces showed interests.
2	(−) *Appreciate*	(I) Paint & narrative	Constructing lived self-identity (Day 1, 70′): Ms painted themselves (*yin*) with their influential past experiences and characteristics associated with these experiences.	Ms reported a deeper self-understanding and higher self-confidence.
3	(−) *Appreciate*	(I) Narrative with our kun story	**Reflecting on our** ***kun*** **story** (Day 1, 100′): L presented pictures and narrated *kun* story (*yin*). Ms identified with one of the six *kun* mare statuses and related it to their job-hunting experience (*yin*).	Ms felt relieved with the sense of universality. They reported a dialectic perspective about job-hunting and accepted their situatedness.
4	(−in+) *Adapt*	(I) Portray	**Inspiring group power to transform job-hunting frustration** (Day 2, 50′): Ms split into groups based on their *kun* mare status.” Each group portrayed its “job-hunting situatedness” (*yin*).	Ms' pictures revealed a dialectic perspective.
5	(−in+) *Adapt*	(R+I) Analyze with our *tai-ji* diagram & narrative	**Inspiring individual power to transform job-hunting frustration** (Day 2, 50′): Ms checked up their individual power to transform frustration with a *tai-ji* diagram (*yin*), within which the white body of fish symbolized individual capacities, black eyes within white body environmental risks, the black body of fish individual weakness, and white eyes within black body environmental resources (*yang*).	Ms identified both positive and negative effects of their own resources and risks in job-hunting. Such learning made a positive change in their evaluation of individual power.
6	(+) *Realize*	(I) Narrative with our *qian* story	**Reflecting on our** ***qian*** **story** (Day 2, 80′): Our *qian* story was processed in a way similar to our *kun* story (*yin*).	Ms grasped the dialectic nature and claimed to take action.
7	(−in+) *Adapt*	(I+R) Narrative with Analyzing	**Making a sequel (*****yin*****) to finish group transformation of job-hunting frustration** (Day 3, 50′): The two groups switched their drawing and worked on the other group' situatedness (-*yin*). Ms identified the protagonist's capacities and strategies as well as highlighted his/her transformative actions (*yang*).	Both *yang* and *yin* power and the principle of “*joint production ”* were demonstrated in their sequel.
8	(+in−) *Realize*	(I+R) Portray and analyze	**Portraying a career vision and constructing occupational strengths** (Day 3, 50′): Ms portrayed their career visions (*yin*) and checked out their readiness for the targeted occupation (*yang*).	Ms revealed clearer career visions and improved their knowledge about occupational requirements.
9	(+) *Control*	(R+I) Plan and announce	**Making an action plan for ideal occupation** (Day 3, 30′): Ms developed and declared their action plans (*yang*) to actualize their occupational goals (*yang*) on stage; other Ms did strong-point-bombing in response (*yin*).	Ms declared specific occupation goals and action plans firmly.
10	(+in−) *Realize*	(R) Rehearsal	**Practicing resume-writing and job interviews** (Day 3, 50′): Ms in pairs took turns to play interviewee and interviewer for a complete job interview (*yang*).	Ms got a better idea about what job interviewers required and improved their self-promoting skills.
11	(+with−) Synthesize	(R) Conceptualize	**Reviewing and synthesizing group learning** (Day 3, 10'): L reviewed their group process and related it to the VEC concepts of *yin-yang* fluxion.	Ms reported what they could think and do differently in the future. They were eager to move on and to take a try.

### Constituent themes

The constituent themes emerged from situated structure analyses of member experience are presented in Table 5. There were five clusters of themes. (1) Sense of situation (SS) described Ms' perception about their career development status. (2) Acceptance (Ac) referred to Ms' attitude toward their developmental status. (3) Self-efficacy (SE) indicated Ms' confidence about their power to unfold their career. (4) Actualization (At) pointed to Ms' planned or actual actions to unfold their career. (5) Dialectic thinking (DT) signified Ms' perception about the dialectic nature of career evolution. The SS themes were revealed in pre-group interviews; the others within group process.

**Table 5:** **Constituent themes of VEC group experience**.

**Cluster**		**G**		**S**		**Theme**
		**Type**	**Step**	**Type**	**Step**	
SS	1		0			G: I am not sure where I am in the world.
SS	2				0	S: I am stranded and depressed.
SS	3		0		0	G: I am not sure where I can go. S: I see no way out.
Ac	1	+	1~4			G: I feel comfortable with uncertainty.
Ac	2	±	15~16	−	3~5	S: I accept my current situatedness (what I encountered now). G: I will accept whatever I encounter.
SE	1	−	10~12	−	1~2	G: I see how I struggled and survived in my past. S: I see how I suffered and survived in my past.
SE	2	−	10~12	−	3~5	G+S: I believe I have more power than I thought.
SE	3	−	10~12	−	1~2	G+S: I believe in my power to survive in the future workplace.
SE	4	+	5~9			G: I see how to use my power to meet future challenges.
At	1	+	1~4	+/±	8~11	G: I get an accessible career vision. S: I get a clearer career vision.
At	2	+	1~4	+/±	8~11	G+S: I see my targeted job (destination).
At	3	+	1~4	+/±	8~11	G+S: I see what my targeted job (destination) requires.
At	4	+	5~9	+/±	8~11	G+S: I see how to prepare myself for my targeted job (destination).
At	5	+	13~14	+/±	8~11	G+S: I see how to promote myself for my targeted job (destination).
At	5			+/−	6~7	S: I decide to take actions.
At	6			+/±	8~11	S: I am eager to take actions.
DT	1			−	3~5	S: I see the dialectic nature of career power (risks and resources).
DT	2			−	3~5	S: I see the dialectic nature of job-hunting (projecting strategy).
DT	3			+/−	6~7	S: I see the dialectic nature of career success.
DT	4	±	15~16			G: I see the dialectic nature of career evolution (VEC model).
DT	5	±	15~16	+/−	6~7	G: I synthesize all my positives and negatives to find an ideal Self and career vision. S: I use the dialectic nature of my power to find a way out.

*SS, sense of situation; Ac, acceptance; SE, self-efficacy; At, actualization; DT, dialectic thinking; G, growing college students; S, stranded college graduates; +, yang type strategies; −, yin type strategies; ±, synthesized strategies*.

### Situated structure

**Figure 3:**
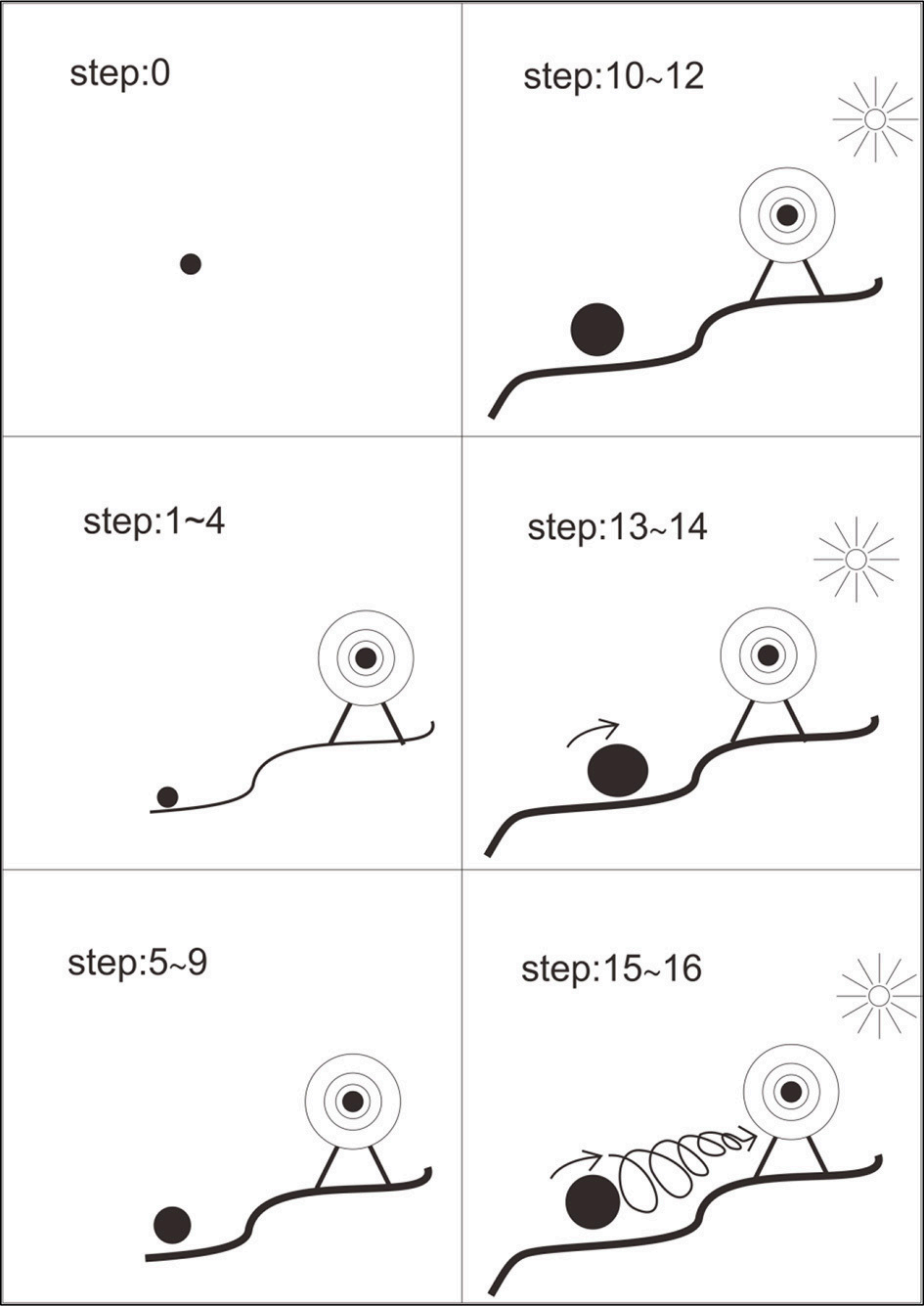
**Growing developers' transformative experience during group process**.

Figure 3 shows growing developers' transformative experience during group process. At the beginning, they stood along with no past or future since they didn't know where they were and where to go. At step 1~4, with *yang* strategies and our *qian* story, they got an accessible future with a clearer career vision and a vague path toward their target. They felt more comfortable with uncertainty after step 1. They moved on and worked on their career visions, which resulted in a better sense of what they wanted from the world and what they needed to supply to the world. At step 5~9, with *yang* strategies and our mind map technique, their future became concrete. They personalized their occupational strengths and went into details about their learning plans and job-hunting preparation, which resulted in self-efficacy. At step 10~12, with *yin* strategies and our *kun* story, they got a powerful past, that is, lived workplace strengths applicable to future workplace adaptation and socialization. Their self-efficacy increased with a continuity from their past to their future. At step 13~14, with *yang* strategies, they got an action-oriented now. Their self-promoting skills were improved via practicing. At step 15~16, with *yang* strategies and mandala drawing, they depicted a cyclical-spiral action process toward future.

**Figure 4:**
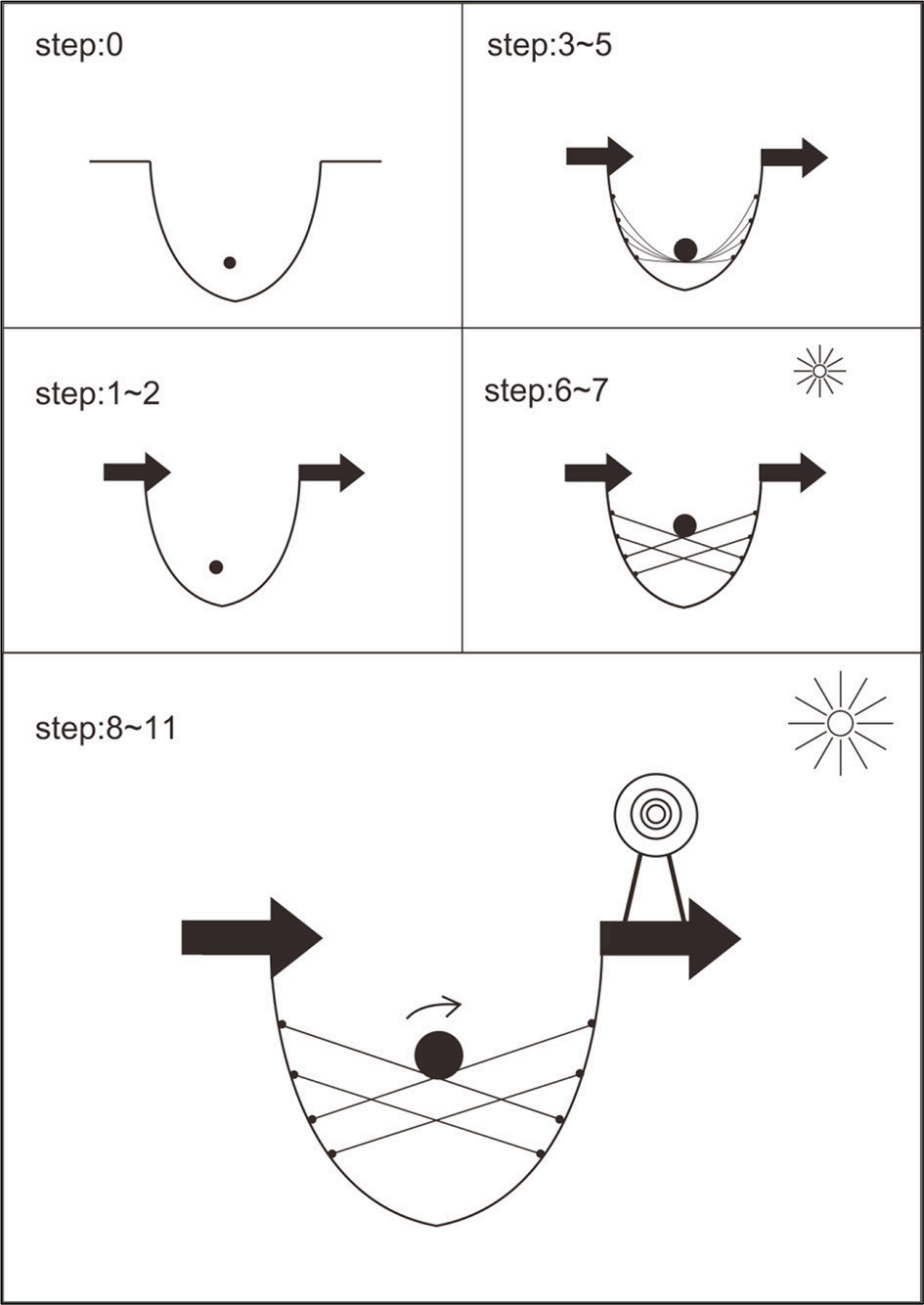
**Stranded developers' transformative experience during group process**.

For stranded developers (see Figure 4), at the beginning, they stuck in a hole since they saw no way out. At step 1~2, with *yin* strategies, they got a valuable self. Via their lived past, they were moved and empower. At step 3~5, with *yin* strategies and our *kun* story, they got a workable now with a dialectic perspective. They accepted their current situatedness and found out their individual power to transfer their job-hunting frustration. At step 6~7, with *yang* strategies and our *qian* story, they got a symbolic working-through experience with a dialectic perspective. Their self-efficacy and dialectic problem-solving skills increased, which resulted in their motivation to take action for their real-life challenges. At step 8~11, with *yang* strategies, they got a workable future with a clearer career vision and a concrete path toward their target. They came out with what they could think and do differently for their real-life situatedness.

### *Yang* and *Yin* strategies for the growing and the stranded

An examination of Table 5 reveals differential group experiences associated with *yang* and *yin* strategies. Although, not exclusively, y*ang* type of career praxes tended to induce actualization, which resulting in realistic goals and concrete action plans; *yin* type of career praxes tended to increase self-efficacy, which resulting in positive attitude toward current situatedness and future development. Acceptance and dialectic thinking often resulted from a blending of *yin* and y*ang* type of career praxes.

More importantly, a comparison of Figures 3 and 4 indicates the growing and the stranded developers need a different arrangement of career strategies. At the beginning, for those growing developers missing a context, CA-*controlling* or CA-r*ealizing* (*yang* power) helps to make their goals clear and put their strengths together. For those stranded developers suffering, CA-*appreciating* or CA-*adapting* (*yin* power) helps to stop complaining or giving up, to come down, and to make up their mind to start with whatever they have. In the middle, a switch of *yin* and *yang* types is needed to create harmonization. For those growing developers, subsequent CA-r*ealizing* or CA-*adapting* or CA-*appreciating* helps to make them humble and open-minded. For those stranded developers, subsequent CA-*adapting* or CA-r*ealizing* or CA-*controlling* helps to make them strong and focus. Both growing and stranded developer needed to have CA-r*ealizing* and CA-*adapting* between CA-*controlling* and CA-*appreciating* to bring *yin*-*yang*-mixed power and transformative strategies into their career evolution. CA-synthesizing at the end blended *yang* and *yin* power together, created a sense of integrity, built up a broader framework of career evolution, and strengthened both *yin* and *yang* power to be applied in the future.

### Experience of our metaphorical stories

The stranded developers revealed the following learning from our *qian* story. (1) One should keep hope and goal for one's life and never give up for learning or trying (S2 and S8). (2) One should build up one's strengths before setting off (S8). (3) One should accept others' help when needed (S11). (4) One should not get over-excited when things go well (S3 and S7). (5) Career evolution, like any other kind of human growth, unfolds step by step (S9). (6) Life goes on no matter what's absent. (7) You never know you may fly in the sky as a dragon someday. Ms also grasped the dialectic nature. S1, S2, S3, and S9 mentioned “goals could emerge from frustration” and “resources could be found within obstructs.” S5, S6, S7, S8, and S11 mentioned “caution should be maintained after success.” They claimed to take action instead of worrying about possible difficulties or problems. The growing developers failed to follow L's request to identify with the flying dragon. Nevertheless, they reflected on how and why the protagonist dragon could swim under the water and fly in the sky.

As to our *kun* story, the growing developers learned the following. (1) The *kun* mare needs to find their right masters; one needs to find one's right occupations (G5). (2) One can't identify one's strength and weakness unless one sets up a specific goal (G5). (3) Knowing one's strength and weakness is the starting point of current occupational preparation (G1, G2, G6, and G7) as well as the base for future workplace adjustment (G4). (4) One should calm down and use what one has creatively as well as improves what one has progressively (G3). The stranded developers learned the following. (1) “One should live within one's means and not go to inappropriate places (S3, S7).” (2) “One should improve one's skills first so as to seize opportunities (S2).” (3) “One should live in the present (S2).” (4) “Problems could be solved via circumventing obstruction (S8).” (5) “Frustration should be faced with a firm stand (S4).” (6) “One should adjust oneself even if life was not friendly (S2 and S5).” (7) “One should choose a rightful environment (S3, S6, S7, and S10).” (8) “One should appreciate, respect, and pardon everyone (S1).” Ms also expressed that they felt relieved and supported for “having other Ms as companions in the same boat.”

In sum, experience difference between our *qian* and *kun* stories was vague. Both stories helped developers to grasp the dialectical nature of *yin-yang* fluxion. They reframed themselves and their surroundings so as to find out versatile perspectives of managing their situatedness, which was helpful for their secular goal of *living on the earth with ways and harmony*.

### VEC concepts learning from experiential counseling activities

According to Ms' products and feedback, the followings are of special value to deliver VEC concepts. They induced Ms' reflective attitudes and opened the door to the *yin-yang* fluxion principles and a new perspective of their situatedness.

Via portraying individual power *tai-ji* diagram for job-hunting situatedness, the stranded developers revealed their learning of the VEC concept of “*completion and enhancement.”* For example, S3's good quality of “being good at observing others and environment” was found to have a negative effect of “being oversensitive and hesitating in any action.” S1 found that his so-called risk of “low confidence” actually had a positive effect of “easy going.” Such learning made a positive change in their evaluation of individual power.

Via constructing lived workplace strengths, the growing developers revealed the followings. (1) “I appreciated my life; whatever happened in my life was not a waste.” (2) “Capacities used to deal with one's past life events could be transferred to adaptation and socialization in the future workplace.” (3) “Even ‘bad’ thing could be worthwhile.” (4) “I became much more confident in my workplace adaptation and socialization since what I have demonstrated or learned in my past life was much more than I thought.” In sum, Ms grasped the VEC concepts of *mutual completion and enhancement* and *joint production*.

Via practicing “meaning-making game in life field,” the growing developers revealed the following learning. (1) Change is inevitable in real life (S1). (2) One should appreciate and live with whatever happens (S1). (3) Obstructions can be overcome, adjusted, circumvented, transformed, or made use of (S3 and S6). (4) Unexpected stuff could create *inclusive opposites* and generate the power and energy needed for a harmonizing process (S4, S8, and S11). (5) One should accept what couldn't be changed at this moment and wait for a good timing or opportunities for transformation (S8, S10, and S11). (6) One should be responsible for making one's life meaningful to and loved by oneself (S1 and S3). Ms grasped the essentials of our *kun* story, the *generation by opposition* principle and the nature of harmonizing.

Via making a sequel to “job-hunting situatedness,” the stranded developers demonstrated their learning of the principle of “*joint production of yin* and *yang”* in their sequels. The sequel to “where's the road” turned out to be “Move on, young man.” The sequel to “invisible support” was “visible obstructs.”

Via practicing harmonizing with mandala drawing, the growing developers presented their positive-negative-coexistence in a creative and harmonious way. Here were several examples. (1) G4 distinguished his positives and negatives clearly and placed them in separate areas within his mandala circle. However, he was surprised that those positives and negatives pieces formed a big peace symbol. (2) G8 placed his essentials in an umbrellas shape. He brought out a story that: “One should remain curious. If encountered something bad, one just learned to probe with curiosity.” (3) G6 put a shining sun in the center, light blue sky on the upper half, and deep blue ocean on the bottom. His story was that: “A bright sky might be an illusion; just like one's limit might be imagination. Both light and dark were parts of oneself. One should explore widely and develop oneself at a good timing in order to bring one's potential to full play and realize one's version.”

## Contributions and limitations

The purpose of this research is to complement western modernism and postmodernism career theories with eastern Chinese wisdom derived from the *Classic of Changes*. Based on thorough theoretical analyses and an action research, we construct a VEC model and counseling prototype, aiming at a secular goal of *living on the earth with ways and harmony* as well as an ultimate end to spiral up to the *wisdom of living up to the way of heaven with mind and virtue*. There are five major career strategies to constitute a VEC counseling process. Situated structure analyses revealed primary empirical support for our model and prototype. In the context of developing indigenous career theories and practices in a globalized and ever-changing world, our contributions appear to be the followings.

1. Echoing pluralism advocated by postmodernism, we propose a concrete career model to blend the virtues of modernism and postmodernism. Postmodernism emerged as the forces of reaction to modernism. As predicted by the *yin-yang* philosophy (Cheng, 2009a), such contradictoriness between these two paradigms did create the power of harmonization for the evolution of career literature. Our model and prototype present an important example which takes advantage of both paradigms and results in harmony.
2. Derived from dialectical philosophy, *w*e built up a complete *yin-yang* continuum of career praxes. In addition to *Controlling* (with pure *yang* power) and *appreciating* (with pure *yin* power), we include *realizing* and *adapting* (with *yin*-*yang*-mixed transformative power). Therefore, a broader range of career praxes is brought into play.
3. We enrich the modernism and postmodernism career practice. Instead of plain preaching, a couple of experiential counseling materials and activities are developed to deliver dialectic philosophy. Two metaphorical Stories and several experiential activities guide participants to think or act in a dialectic way by unfolding the other sides, including the opposites, or making a dramatic extension or turn.
4. We enrich the Middle Way literature. The *yin*-*yang*-mixed strategies are the apple of the Middle Way philosophy, which has produced fruitful and substantial influences in the indigenous psychology rooted in Confucian philosophy (e.g., Huang et al., 2012; Chou et al., 2014). Nevertheless, the Middle Way literature has little production in career theories or practices. Our model and prototype help to supply this gap.
5. We extend the Middle Way literature by proposing specific arrangement principles. The Middle Way tends to focus on those strategies with *yin-yang*-mixed transformative power only; it fails to include strategies with strong *yin* or strong *yang* power. Our arrangement principles help to combine various strategies in a dialectical and harmonious way.
6. Echoing second wave positive psychology and virtue psychology, we extend the mixture of modernism and postmodernism by embracing the spiritual/ethical essential of career evolution. With Confucian philosophy emphasizing both horizons, our model and prototype set up relevant conceptualization and practices to connect the secular and spiritual/ethical horizons in the process of career evolution.

To establish an alternative career theorization and practices for eastern and western people in a globalized and ever-changing world, the VEC model and prototype need further elaboration. As indicated by *yin*-*yang* philosophy, the limitation of our model and prototype lie on the other sides of their contributions. Serving as a meta-theoretical framework, our model and prototype possess great depth and wisdom, which also result in a need for more practical details. Also, future research should extend its applications to various career issues or phenomenon. Finally, standardized control studies are needed to further verify our breaking points.

## Funding

This research project was granted by Ministry of Science and Technology of R.O.C.'s funding (NSC102-2410-H-034-005-; MOST 103-2410-H-018-008-; MOST 104-2410-H-018-010); we thank for the support.

## Author contributions

SL was the leader of this project. Her major contribution was the conceptualization and design of the project, interpretation of data, and writing this report. JH was co-leader of this project. His major contribution was the conceptualization of the project and interpretation of data. HP was the executor of counseling groups. She also helped to analyze data and wrote stories. CC was the literature organizer and help with group observation and data analyses. YL was the data interpreter.

### Conflict of interest statement

The authors declare that the research was conducted in the absence of any commercial or financial relationships that could be construed as a potential conflict of interest.
